# Short term results of anterior cruciate ligament augmentation in professional and amateur athletes

**DOI:** 10.1007/s10195-017-0447-x

**Published:** 2017-02-13

**Authors:** Hamidreza Yazdi, Ali Torkaman, Morteza Ghahramani, Amin Moradi, Ara Nazarian, Mohammad Ghorbanhoseini

**Affiliations:** 1 Orthopaedic Surgery, Department of Knee Surgery, Firoozgar Hospital, School of Medicine, Iran University of Medical Science, Tehran, Iran; 2 Department of Knee Surgery, Shafa Rehabilitation Hospital, Iran University of Medical Science, Tehran, Iran; 3 Orthopaedic Surgery, Nazarian Lab, Center for Advanced Orthopaedic Studies, BIDMC, Harvard Medical School, Biomedical Engineering, Boston University, 330 Brookline Ave., RN 115, Boston, MA 02215 USA; 4 Harvard Medical School, 67 Park Street, Brookline, MA 02446 USA

**Keywords:** ACL augmentation, Posterolateral bundle, Anteromedial bundle, ACL tear, Athletes

## Abstract

**Background:**

Anterior cruciate ligament (ACL) reconstruction is a widely accepted procedure; however, controversies exist about ACL augmentation. The purpose of this study was to assess the clinical outcomes of ACL augmentation in professional and amateur athletes with isolated single bundle ACL tears.

**Materials and methods:**

A consecutive series of professional and amateur athletes with partial ACL tears who underwent selective bundle reconstruction were analyzed. Stability was assessed with the Lachman test, anterior-drawer test, pivot-shift test and KT-1000 arthrometer. Functional assessment was performed using the subjective Lysholm questionnaire.

**Results:**

Fifty-six patients were enrolled. The mean follow-up period was 19.3 months. All patients had posterolateral bundle (PLB) tears, and no anteromedial bundle (AMB) tears were found. The Lysholm score improved significantly from 78 (SD = 2.69) preoperatively to 96 (SD = 3.41) postoperatively (*P* value <0.0001). The pivot-shift test, Lachman test and anterior-drawer test results were negative in all cases postoperatively. Anterior tibial translation from neutral was 4.9 mm (SD = 2.7) preoperatively, and decreased significantly to 2.1 (SD = 0.6) postoperatively, measured with a KT-1000 arthrometer (*P* value <0.00001).

**Conclusion:**

In this study, we showed that ACL augmentation had good results in symptomatic professional and amateur athletes, and although further studies are needed to investigate long-term results, we recommend this surgery for all symptomatic athletic patients, especially those who would like to maintain an active lifestyle.

*Level of evidence* IV.

## Introduction

Each year 80,000–250,000 anterior cruciate ligament (ACL) injuries occur in the United States, with the majority occurring in athletes between 15 and 25 years of age [1–4].

Partial tears of the ACL, although not as common as complete tears, may account for 10–28% of all ACL injuries; however, epidemiologic data on partial tears is not as clearly defined [3, 5]. Mott first described the surgical reconstruction of the ruptured fibers of the ACL while preserving its remnants three decades ago [3]. He defined it as an “ACL augmentation technique”. However, this technique has become more popular in the last few years, as the double bundle ACL reconstruction technique has started to be used more frequently [6].

Preserving the uninjured bundle has a number of theoretical advantages [6–15]. ACL remnants may add biomechanical strength to the reconstruction in the immediate post-operative period, while graft strength depends primarily on the fixation device [7–10, 16, 17]. Moreover, the residual portion of the ACL may maintain its blood supply, and thus provide support to the healing process of the graft [9, 11, 12, 16, 18, 19]. Maintaining some of the proprioceptive innervation of the ACL might allow for a faster return to sports [9, 11, 14, 20]. Finally, the intact bundle might help to optimize the accuracy of bone tunnel placement by serving as a landmark [21].

The purpose of this study was to assess the clinical outcomes of ACL augmentation in athletes with isolated single bundle ACL tears. To that end, the hypothesis was that subjective and objective outcomes would improve significantly with arthroscopic ACL augmentation.

## Materials and methods

Fifty-six professional and amateur athletes with partial tears of the ACL were enrolled in this project from June 2009 to August 2012. The inclusion criteria were history of trauma (direct or indirect) and partial ACL tear, all in a symptomatic athlete. The exclusion criteria were complete ACL tears, multiple ligament injuries, nonathletic patients, significant malalignments in need of correction, asymptomatic patients referred only for MRI findings and injuring the intact bundle during surgery. Chondral lesions and meniscal tears were not considered exclusion criteria, and were addressed at the same operation before augmenting the partial ACL tear. All patients were examined, selected, consented and later operated on by a senior surgeon. Follow up examinations were also conducted by the same senior orthopaedic surgeon.

All participants were male, with a mean age of 24.3 years (range 17–35 years). The average interval from trauma to arthroscopic surgery was 10.2 weeks (range 3–48 weeks). All cases reported that they felt their knee might give away or had difficulty in sports activities. Patients indicated their grievances as “something is wrong in my knee” or “one side is not like the other side while I am exercising”.

There was a pre-op difference between Lachman test results on the injured and uninjured knees of the participants, indicating increased laxity in the injured knee. Anterior drawer test was one to two plus positive in all cases, and pivot shift test was one plus positive in 23 knees and negative in the rest (33 knees). Both knees in all patients were assessed using KT 1000 arthrometer preoperatively. Radiography and MRI studies were conducted in all patients. Radiography results were normal in all patients, and MR imaging studies suggested that a portion of the ACL was intact while the other portion was damaged.

**Fig. 1:**
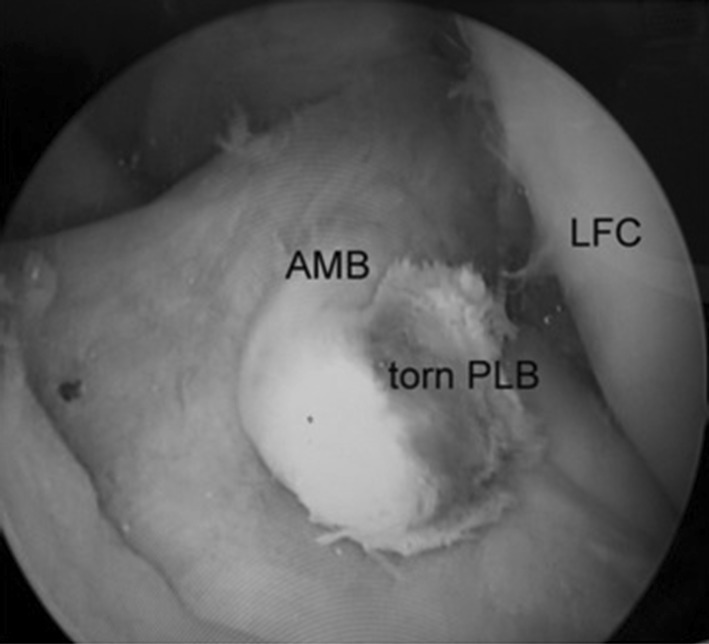
Arthroscopic examination shows single bundle anterior cruciate ligament (ACL) tear. *LFC* Lateral femoral condyle, *AMB* anteromedial bundle of ACL, *PLB* posterolateral bundle of ACL

**Fig. 2:**
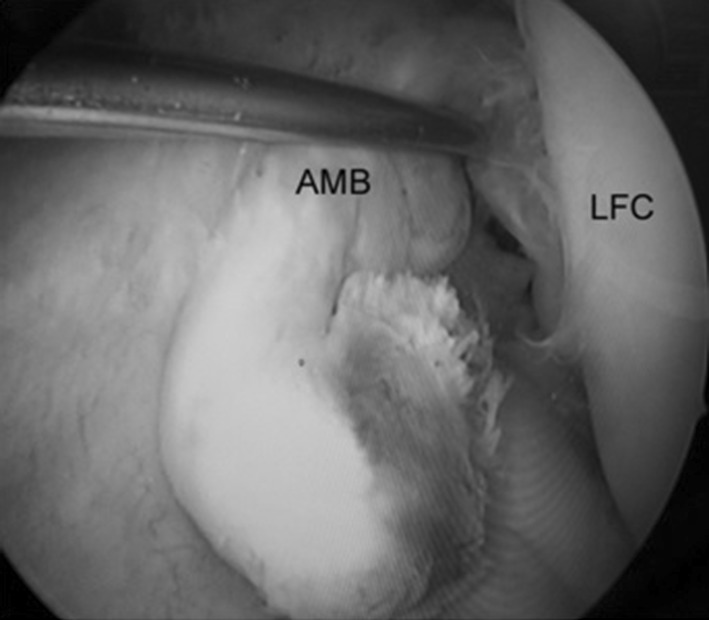
Probing of intact AMB. *LFC* Lateral femoral condyle, *AMB* anteromedial bundle of ACL

All surgeries were performed by a senior orthopaedic surgeon using the same equipment and surgical technique. Preoperative IV antibiotic (Cephazolin—1 g) was administered approximately 30 min before the incision was made. Surgery was done on a universal table. Either general anesthesia or spinal anesthesia was administered by a staff anesthesiologist. The patient was positioned supine, and a tourniquet was inflated on his/her upper thigh. Arthroscopic examination was performed at first using two standard high anterolateral and low anteromedial portals (Fig. 1), where the ACL was probed to evaluate the ligament and verify the partial tear (Fig. 2). All 56 cases had posterolateral bundle (PLB) tears, where the anteromedial bundle (AMB) was visibly and palpably intact. After diagnosing a partial ACL tear, ACL augmentation was performed for the patient.

**Fig. 3:**
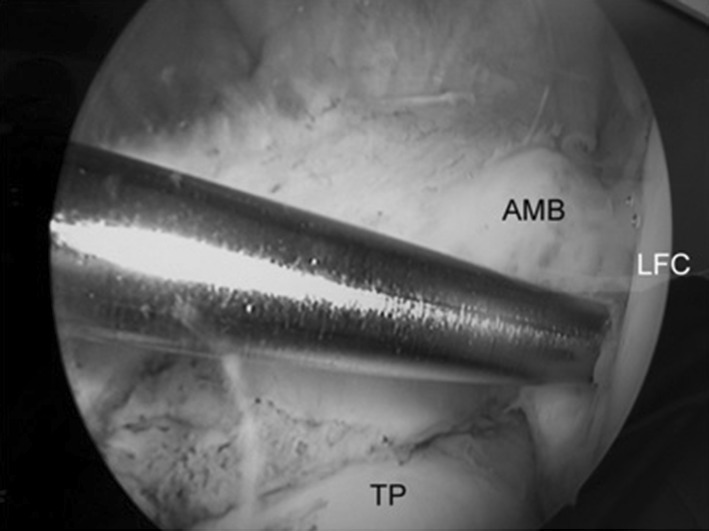
Femoral tunnel positioning using guide wire. *TP* Tibia plateau, *LFC* lateral femoral condyle, *AMB* anteromedial bundle of ACL

**Fig. 4:**
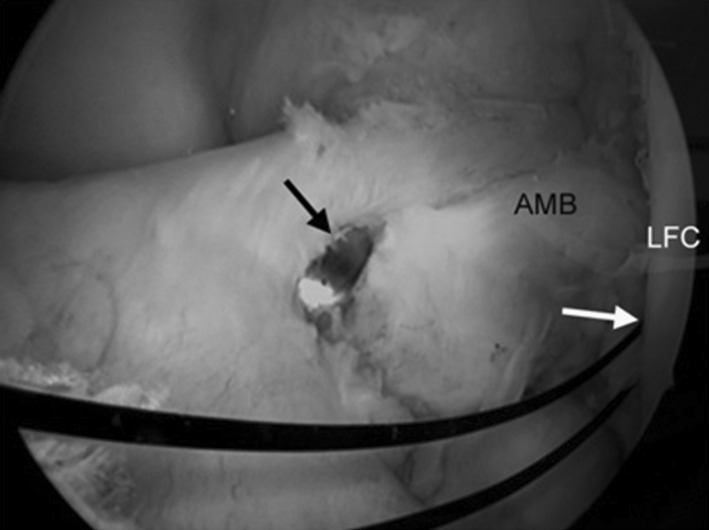
Tibial tunnel positioning. *Black arrow* Tibial tunnel guide pin, *white arrow* femoral tunnel

**Fig. 5:**
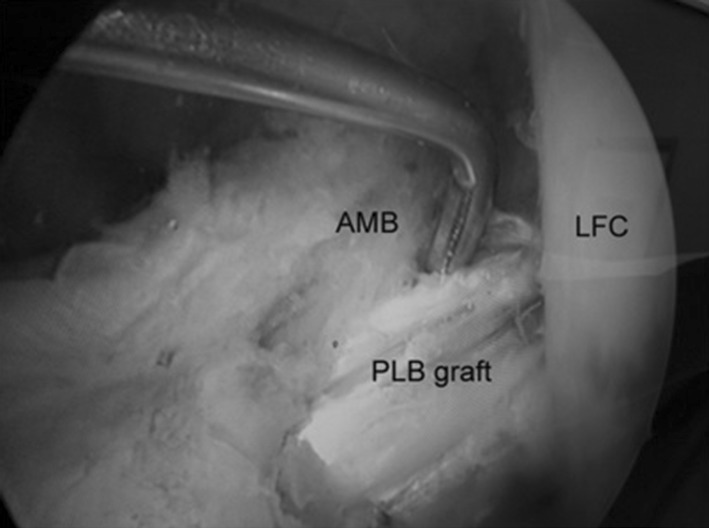
ACL augmented

The semitendinosus (ST) tendon was harvested from each participant, and the graft was prepared to reconstruct the PL bundle. In case of insufficient ST tendon thickness (less than 7 mm), then the gracilis tendon was also used [13]. After locating the femoral (Fig. 3) and tibial (Fig. 4) attachments of the posterolateral bundle, transportal anatomical posterolateral bundle reconstruction was performed (Fig. 5) using a button (Flipptack, Storz, Tuttlingen, Germany) for femoral fixation, and a bioabsorbable screw (Megafix screw, Storz) for the tibial side. Graft fixation was done with the knee in near extension. The anteromedial bundle was preserved during surgery by a retractor, and any meniscal or chondral lesion was addressed before ACL augmentation, where partial meniscectomy was done if necessary. There were no cases of meniscal repair.

A knee immobilizer was used to avoid joint flexion contracture postoperatively. The knee immobilizer was limited to overnight use and while moving about in the public after the 1st day. In the morning after surgery, knee range of motion was initiated between 0 and 45° as tolerated. One day after surgery the patient was discharged from the hospital and was allowed to weight bear progressively as tolerated using crutches. Also doing exercises at home were encouraged, which were described at the hospital. After 2 weeks, full weight bearing was allowed and physical therapy was continued. Return to non-competitive sports was allowed 3 months post-op, and competitive sports were allowed 6 months post-op.

All patients were reassessed immediately after surgery and at each follow-up at 2 weeks, 6 weeks, 12 weeks, 6 months and 1 year after surgery. Lachman and Anterior Drawer Test were checked at each follow-up. At the 1 year post-op visit, arthrometric assessment using a KT-1000 arthrometer and patient satisfaction rate and Lysholm score were also recorded.

Subjective and objective measures were analyzed preoperatively and at a minimum 1-year follow-up. The Independent Student’s *t* test and Chi square test were used for statistical analysis. A *P* value <0.05 was considered significant. All tests were analyzed using SPSS version 16.0 (IBM-SPSS, Armonk, NY).

## Results

The average and median follow up times were 19.3 months (range 12–37 months) and 24 months, respectively. No patient was lost during follow up, and the subjects included 56 male patients with an average age of 24 years old (range 17–35 years). The average interval between injuries to surgery was 10.2 weeks (range 3–48 weeks). All patients had a partial tear in the posterolateral bundle of the ACL. Medial meniscus tear was detected in 15 patients, and lateral meniscus tear was detected in 7 patients. None of the patients had both medial and lateral meniscus tears simultaneously.

The mean Lysholm score improved significantly from 78 (SD = 2.69) preoperatively to 96 (SD = 3.41) postoperatively (*P* value <0.0001). Satisfaction rate was excellent in 42 patients and good in 14 patients. Before surgery, the Lachman test was positive in all patients, whereas the same test became negative in all patients post-operatively. Pivot shift test was one plus in 23 knees and negative in the rest (33 knees) preoperatively and became negative in all patients after surgery. Anterior drawer test was one to two plus positive compared to the other knee in all cases preoperatively. After surgery, it became same as other knee in all cases. Anterior tibial translation from neutral was 4.9 mm (SD = 2.7) preoperatively, and decreased significantly to 2.1 (SD = 0.6) postoperatively, measured with a KT-1000 arthrometer (*P* value <0.00001). All patients had full knee range of motion postoperatively. The only complication we encountered was a superficial donor site infection, which was treated by wound care and oral antibiotic therapy.

## Discussion

We conducted a study on 56 professional and amateur athletes, where we performed ACL augmentation on partial tears from June 2009 to August 2012. We checked anterior drawer test, pivot shift test, Lachman test and KT-1000 arthrometer measurement before and after surgery 1 year post-op. Also we checked subjective results after surgery at that moment. All patients regained their knee range of motion post-operatively, and reported good or excellent results. The mean Lysholm subjective score improved significantly after surgery (*P* value <0.00001), and anterior drawer test, pivot shift test and Lachman tests showed normal results after surgery. Average anterior tibial translation from neutral, measured with a KT-1000 arthrometer, decreased significantly from baseline (*P* value <0.00001). Based on these results, we recommend ACL augmentation for all symptomatic athletic patients.

It is generally believed that ACL does not behave as a simple band of fibers with constant tension [22–24]. Division of this ligament into anteromedial (AM) and posterolateral (PL) bundles, based on insertion of each individual bundle to the tibial surface, is now widely accepted as the basis of understanding of ACL function [22–24]. The AM bundle mainly restrains anterior tibial translation in more than 45° of knee flexion [25–27], whereas the PL bundle has been shown to be less isometric, and a more important restraint towards full knee extension [25, 28]. In addition, PLB has a crucial role in the rotational stability of the knee joint [22, 29].

Typically, patients who sustain a complete ACL tear report symptomatic instability with pivoting in sports or strenuous activities [3]. Patients diagnosed with ACL partial tears have a less predictable outcome. Although many continue to experience instability, some do not, and identifying both groups can be challenging [3]. Moreover, as there are no clear guidelines, diagnosing partial tears and tailoring treatment for individual patients can be difficult [3, 25].

Sensitivity of MR imaging for detecting complete ACL rupture is 94.5% (95% confidence interval 0.92–0.96), and its specificity is 95.3% (0.93–0.97) [30]. However, diagnosis of a partial ACL tear remains a difficult challenge. It is based on clinical examination, radiological and MR imaging studies, but the definitive diagnosis is made by arthroscopic evaluation [8, 16]. The accuracy of standard imaging might be as low as 25–53% for diagnosing a partial ACL tear [31]. An accurate arthroscopic assessment performed by an experienced surgeon is currently considered the best means to confirm the diagnosis [11, 16].

Theoretically, an isolated AM bundle tear would result in a severely positive anterior drawer test and a mildly positive Lachman test with a negative pivot-shift test [25, 32]. In contrast, a patient with a symptomatic PL bundle rupture would complain of the feeling of giving way or instability, which are due to abnormal rotational instability, also there would be a mild anterior instability and a clearly positive pivot shift test [25, 32, 33].

Disease natural history studies have demonstrated that fewer than 50% of patients return to their preinjury activity level [3]. Several studies have also documented that progression to complete rupture is a common complication for patients who return to an active lifestyle [3]. That is the reason why surgical intervention or ligament reconstruction should be considered in highly active patients. The treatment choices for these patients would be ACL reconstruction and ACL augmentation. Several studies have shown that the outcomes after ACL augmentation are superior compared to ACL reconstruction in terms of proprioception and joint stability [7–11, 14, 15, 17]. Saving ACL remnants during ACL reconstruction may have some biomechanical, vascular, and proprioceptive advantages for the patient [9, 11, 14, 16, 30]. ACL remnants add biomechanical strength to the reconstruction in the immediate post-operative period, while graft strength depends primarily on the fixation device. In this period, the augmentation would be protected by the intact remnants and allow accelerated rehabilitation and an earlier return to sports [9, 11–15, 20]. A second important advantage of saving fibers is that the residual portion of the ACL maintains its blood supply, providing support for the healing process in the graft [9, 11, 16]. Saving ACL fibers may also maintain some proprioceptive innervations, which would allow for faster and safer return to sports [9, 11, 14, 16]. Finally, the intact bundle serves as a guide for orientation and a point of reference for the proper placement of the graft as described by Siebold and Fu [21].

Interestingly, all patients in the current study had isolated PL bundle injuries, whereas previous similar studies reported that AM bundle injuries were more common than PL bundle injuries [8, 11, 22, 23].

Eriksson stated that, in his experience, patients with isolated AM bundle ruptures often did quite well and did not need surgery. On the other hand, those with isolated PL bundle ruptures reported subjective instability [22]. In our study, all patients had PL bundle ruptures and also reported feelings of instability or difficulty in the injured knee.

Buckley et al. evaluated 25 patients with partial ACL tears at intermediate follow-up (minimum of 18 months), and found that 60% had good or excellent results. Only 44% of patients resumed sports activities at their pre-injury levels, and 72% reported activity-related symptoms [34], whereas in our study all patients reported good and excellent results. Knee ROM returned to normal in all our cases, and objective measurements including anterior drawer test, pivot shift test, Lachman test and KT-1000 arthrometer measurements, improved significantly to near normal levels.

A potential limitation of this study was that we assumed that the only definitive way to make the diagnosis was to probe the ligament by an experienced surgeon; therefore, we discarded physical exam and MR evaluations as non-accurate modalities. We have to understand this is a subjective finding. Also, it is impossible to determine the exact status of numerous fibers within the apparently intact bundle of the ACL. There is no precise way at the moment to speculate how much of the ligament is ruptured, so patients with partially ruptured ligaments may vary in terms of percentage of ruptured fibers in the ACL.

While we did not directly compare our technique with reconstruction techniques applied for complete ACL tears, the outcomes validated the results of previous studies noting that ACL augmentation could restore nearly normal anterior translation laxity of a partially ACL-deficient knee [7–9, 15, 35–37].

Many studies have shown that most partial ACL tears end in complete tears [25, 33, 38–40], where nonoperative treatment would potentially not satisfy professional athletes who are not willing to limit their level of activity.

In this study, we showed that ACL augmentation had good results in symptomatic professional and amateur athletes, and although further studies are warranted to investigate long-term results, we can recommend this surgery for all symptomatic athletic patients, especially those who would like to maintain their active lifestyle.
